# “Being a Person of Color in This Institution Is Exhausting”: Defining and Optimizing the Learning Climate to Support Diversity, Equity, and Inclusion at the University of Washington School of Public Health

**DOI:** 10.3389/fpubh.2021.642477

**Published:** 2021-04-15

**Authors:** Marie-Claire Gwayi-Chore, Erika Lorenzana Del Villar, Lucia Chavez Fraire, Chloe Waters, Michele P. Andrasik, James Pfeiffer, Jennifer Slyker, Susan P. Mello, Ruanne Barnabas, Elba Moise, Renee Heffron

**Affiliations:** ^1^Department of Global Health, University of Washington, Seattle, WA, United States; ^2^Harborview Medical Center, Seattle, WA, United States; ^3^Vaccine and Infectious Disease Division, Fred Hutchinson Cancer Research Center, Seattle, WA, United States; ^4^Department of Anthropology, University of Washington, Seattle, WA, United States; ^5^Department of Epidemiology, University of Washington, Seattle, WA, United States; ^6^Department of Medicine, University of Washington, Seattle, WA, United States; ^7^College of Education, University of Washington, Seattle, WA, United States

**Keywords:** learning climate, diversity, equity, inclusion, antiracism, racism, homophobia, sexism

## Abstract

Learning climate greatly affects student achievement. This qualitative study aimed to understand community definitions of climate; share lived experiences of students, faculty, and staff; and define priority areas of improvement in the University of Washington School of Public Health (UWSPH). Between March-May 2019, 17 focus group discussions were conducted–stratified by role and self-identified race/ethnicity, gender and sexual orientation–among 28 faculty/staff and 36 students. Topics included: assessing the current climate, recounting experiences related to roles and identities, and recommending improvements. Transcripts were coded using deductive and inductive approaches. Race/ethnicity, gender, and sexual orientation appeared to affect perceptions of the climate, with nearly all respondents from underrepresented or minoritized groups recounting negative experiences related to their identity. Persons of color, women, and other respondents who identified as lesbian, gay, bisexual, transgender, queer/questioning, intersex, and asexual (LGBTQIA) frequently perceived the climate as “uncomfortable.” Most felt that UWSPH operates within a structural hierarchy that perpetuates white, male, and/or class privilege and “protects those in power” while leaving underrepresented or minoritized groups feeling like “the way to move up… is to conform” in order to not be seen as “someone pushing against the system.” Improvement priorities included: increasing community responsiveness to diversity, equity, and inclusion; intentionally diversifying faculty/staff and student populations; designing inclusive curricula; and supporting underrepresented or minoritized groups academically, professionally, and psychologically.

## Introduction

Among higher education institutions (HEIs), the campus climate is often a critical factor influencing success in recruiting and graduating students, as well as hiring, retaining, and promoting faculty and staff. Campus climate incorporates numerous facets of an institution, including the physical spaces where teaching and learning take place (i.e., classrooms, lectures, libraries, and other on- and off-campus spaces); the resources that produce learning outcomes (i.e., instructors/tutors, curricula, and course materials); the structural diversity and institutional history of an HEI; and the perceptions, attitudes and behaviors of students, faculty, and staff regarding their institutions (1–3). Climate is also defined as a socio-environmental factor that is associated with key cognitive, social, participatory, and attitudinal outcomes of students (2). For the remainder of this paper, we use the term *learning climate*, rather than *campus climate*, to broaden the focus from the location of education to its intention of developing and furthering knowledge and experiences of students, faculty, and staff.

Numerous factors affect the HEI learning climate, including: “its historical legacy of inclusion or exclusion of various racial/ethnic groups; its structural diversity or the numerical representation of various racial/ethnic groups; perceptions and attitudes between and among groups, and interactions; and relationships between diverse campus groups (4).” According to Steele's stereotype threat theory, adverse learning climates negatively affect student academic performance (e.g., course assessments, national examinations, or grade point averages), especially for students of color and other marginalized groups (5, 6). The theory posits that when students are reminded that they belong to a group that is stereotypically defined to be academically inferior, they often perform at lower levels than their counterparts in the majority population due to the pressure of conforming to these stereotypes, despite having similar levels of preparation. Oftentimes, these “reminders” are microagressions–subtle verbal, non-verbal, and/or visual insults directed toward marginalized groups–that are experienced by students of minoritized identities who are repeatedly reminded about their inferiority due to their race, ethnicity, or gender identity.

Today, numerous HEIs conduct annual learning climate assessments that include students as well as other campus populations–namely faculty, staff, and leadership—in order to provide more comprehensive evaluations and develop more inclusive responses to improve overall diversity, equity and inclusion (DEI). In 2008, the University of Washington School of Public Health (UWSPH) conducted its first such climate survey, with subsequent surveys in 2017 and 2018. According to the 2018 results report, the average rating of comfort with the current climate was 3.55 on a 1–5 Likert scale (7). Although the percentage of respondents who felt “comfortable” or “very comfortable” did not substantially change from 2008 (57.4%) to 2018 (58.6%), the percentage who rated the climate as “very uncomfortable” and “uncomfortable” nearly doubled over the same time period (9.9 vs. 18.1%) (7). In addition, persons of color (POC), women, and those from low-income backgrounds consistently reported feeling “uncomfortable,” with POC rating the climate the lowest at 2.91 (7). Among those who reported any level of discomfort, microaggressions and exclusionary behavior were the most common offenses, specifically related to participants' racial, ethnic and gender identity, and sexual orientation. Given the disproportionate feelings of discomfort with the climate among marginalized populations, understanding how experiences with implicit bias and microaggressions affect learning and working in UWSPH is critical to eliminate opportunity gaps based on race, ethnicity, gender, or disability. A limitation of the quantitative climate survey approach is the inability to descriptively capture reasons why respondents defined the climate to be sub-optimal. Thus, this qualitative study was designed to conduct an in-depth examination of faculty, student, and staff perceptions of the current learning climate within the UWSPH, the range of experiences of diverse groups operating within that climate, and perceived areas for improvement in the context of DEI.

We also want to illustrate the lived experiences of people identifying with particular demographic groups [i.e., POC, White, women, and persons identifying as lesbian, gay, bisexual, transgender, queer/questioning, intersex, and asexual (LGBTQIA)] and their perspectives on factors contributing to the climate, including: curriculum, recruitment, retention, and promotion, as well as availability and distribution of campus resources. We also present participants' recommendations of strategies that may improve the learning climate.

## Materials and Methods

### Study Design

We conducted a cross-sectional qualitative study using a phenomenological approach to assess perceptions and experiences of students, faculty, and staff within the UWSPH about the learning climate (8). Focus group discussions (FGDs) with representatives across the broad array of UWSPH departments and programs were conducted to develop a deeper and more nuanced understanding of their experiences, the meanings attached to those experiences, and their suggestions to address cultural and social disconnects that emerged from previous UWSPH Climate Survey data.

### Study Setting and Participants

FGDs were conducted between March and May 2019 within UWSPH, a large public health school located in the Pacific Northwest region of the United States. UWSPH has 40 graduate degree programs across five academic departments and five interdisciplinary programs. There are ~1,700 graduate and undergraduate students, 250 core faculty, and 660 staff who teach, mentor, and provide direct support to students. Approximately 20% of the undergraduate and 15% of the graduate student populations are underrepresented minorities (i.e., African American, American Indian/Alaska Native, Hawaiian/Pacific Islander and Hispanic/Latino), according to the 2017 census data (9). School leadership (i.e., Deans, Department Chairs, or Program Directors), faculty, staff, graduate and undergraduate students within any UWSPH department were invited to voluntarily participate in the study. Participants were offered a $10 gift card for their time.

### Recruitment and Data Collection

Participants were recruited by emails sent through UWSPH listservs, in-person classroom announcements, individual interactions, and print advertisements widely posted around UWSPH common areas. Recruitment continued on a rolling basis until FGDs were conducted with all targeted groups and data saturation was reached. Individuals who expressed interest in participating were invited to complete a confidential online form and asked to indicate their identities, including role (leadership, faculty, staff, or undergraduate/graduate student), race (Black/African American, White, Native American, Alaskan Native, Asian, Hispanic/Latinx, Native Hawaiian, or Pacific Islander), gender identity (female, male, transgender, genderless, non-binary, bigender, third gender, gender fluid), or sexual orientation (homosexual/lesbian, bisexual, or asexual). Respondents were also given an “Other” option for any identity not listed. Based on these identities, respondents selected their top three FGDs of choice. These preferences allowed the researchers to create representative groupings for more robust discussions and to tailor prompts based on each group's shared identity.

Discussion guides were developed separately for faculty/staff and student FGDs and each focused on four major domains: (a) defining the “ideal” learning climate and its effect on learning and outcomes; (b) assessing the current UWSPH climate (rating the comfort of the climate from “very uncomfortable” to “very comfortable”); (c) collecting positive and negative experiences in the context of participant self-defined identities and roles; and (d) recommending actions to improve the learning climate through the promotion of policies and norms that promote anti-racism, anti-sexism, anti-classism, anti-sexual prejudice (i.e., homophobia or transphobia).

FGDs were conducted in private rooms by two external qualitative researchers (ELDV and LCF) who were not current student, faculty, or staff in UWSPH. One facilitator led each 60-min session in English and also served as the note taker. Sessions were audio recorded after participants gave oral consent for recording. Recorded discussions were transcribed by an external translation firm (GMR Transcription, Tustin, California, USA) and underwent quality assurance processes. While all sessions were recorded, two sessions which only had one participant were not transcribed. For these sessions, facilitators recorded detailed field notes to supplement the audio recordings that were subsequently collated and included in the analysis.

### Data Analysis

The analysis included both deductive and inductive coding approaches (10). During the data collection process, both facilitators started developing codes to avoid single-coder bias. They held debriefing sessions within a day or week after completing a series of FGDs to share data and record preliminary themes that emerged from the discussions followed by debriefing meetings with the study principal investigators (M-CG-C and RH). A codebook was then developed, including codes based on these preliminary themes, together with the domains probed in the discussion guides (Supplementary Table 1). The coding process was conducted by a single analyst using Dedoose v.8.0.35 software in two rounds. The first round consisted of coding the transcribed data based on the initial codebook. *In vivo* codes were also created when new themes outside of the original domains emerged and these were added to the codebook. In the second round, the analyst then recoded the transcripts to increase the credibility of the data analysis process and also cross-checked the coded data with researcher field notes, facilitator summaries, as well as audio recordings.

### Ethical Considerations

The University of Washington Human Studies Division determined that this study was minimal risk and exempted from ethical review. The identities of all participants were only known to the FGD facilitators during the recruitment process and participants remained anonymous during data collection. No reference to their names, titles, or roles were made during the FGDs. If such references were accidentally broached during the discussion, these names or titles were redacted during the transcription process. All FGD audio files were stored in a password-protected cloud storage system accessed only by the immediate research team. In order to ensure confidentiality for all participants, all names and identifying characteristics have been anonymized.

## Results

A total of 85 individuals expressed interest in participating in a FGD. Due to attrition from scheduling conflicts and cancelations, 64 participants–28 faculty/staff and 36 students–participated in the study. In total, 17 FGDs were conducted: seven with faculty and staff (including two with school leadership) and 10 with students (Table 1). Although we did not capture individual level demographics for all participants, care was taken to include, as much as possible, a diverse representation across the various identities. Among the faculty, we were able to recruit across professorship rank. Among staff, we recruited from both student-facing and non-student-facing positions. Leadership included departmental and school-level leadership. Given the minority representation of some identities within the school, we chose not to report demographics by identity to protect participants' confidentiality. Broadly, the following ethnic identities were represented based on participant self-identification during FGDs: African, African-American/Black, White, Hispanic/Latinx, Southeast Asian, and Pacific Asian Islander. Similarly, we did not capture sexual orientation but we observed LGBTQIA participants who self-identified as lesbian or queer.

**Table 1:** Type and number of FGDs and participants.

**FGD type**	**Faculty and staff**		**Students**	
	**Session *N* = 7**	**Participants *N* = 28**	**Session *N* = 10**	**Participants *N* = 36**
Persons of color (POC)	1	4	4	17
White/non-POC	1	2	1	4
Women	2	10	3	9
LGBTQIA	1	4	2*	6
Leadership	2	8	n/a	n/a

* *One FGD had a combination of POC and LGBTQIA identities*.

The findings are reported within the four research domains of interest, including: the definition of an ideal climate, the assessment of the current climate, positive and negative experiences within the climate, and recommendation for improving the climate. Additional quotes for each theme are found in Supplementary Table 2.

### The Ideal Climate and Its Importance to Learning and Working

Faculty, staff, and student participants viewed seven major characteristics as priority for an ideal learning and working climate: inclusiveness and belongingness; intentional diversity; respect; physical, emotional, and psychological safety; openness and freedom to express oneself, ask questions, take risks, and make mistakes without judgment; adequate mentorship to nurture learning and career growth; and the presence of physical and social spaces conducive for learning. Across all participant groups, having an ideal climate was perceived as essential or non-negotiable.

[Our] experience should be challenging and rigorous from an academic standpoint, but… [interactions with our teachers or peers] shouldn't necessarily add to that difficulty. It should be facilitating learning and not adding to some type of traumatic experience that the student then has to process through. (Student, POC).

Those who posited that the ideal climate was “very important” (a lesser designation than “essential” or “non-negotiable”), were mostly POC, who shared that they were used to “compromising and adapting to” their current climates. A common theme across all respondents was the effect of climate comfort on learning outcomes.

I think if you could have professors who make you uncomfortable or don't make you feel safe, you [can] succeed in the class by just getting the grade that you need to get. I don't think that it puts you in a position to really succeed outside of the classroom. You're not gonna reach out to the professor for mentorship; you're just gonna go to the class and go home. (Student, POC).

Out of 120 students this year… we failed one student and felt terrible about that because it was a student of color… and we reached out repeatedly. I will never know what failed for that student. I know we failed because she didn't complete the class. I have to believe a part of it was the environment. (Faculty, White).

### Assessment of the Current UWSPH Climate

Three key themes emerged across all respondent groups and across demographic cohorts in regards to their assessment of the current UWSPH learning climate: (a) the effect of race/ethnicity, gender identity, and sexual orientation on comfort within the school, (b) a lack of diversity of faculty, staff, and students across UWSPH, and (c) the opinion that UWSPH is inherently structured with a hierarchy that perpetuates White, male, and/or class privilege.

*1. The effect of race and gender identities and sexual orientation on comfort of the climate*.

Varying levels of comfort were experienced by UWSPH faculty, staff, and students with regards to the overall learning climate at UWSPH. These variations appear to be significantly shaped by one's race/ethnicity, gender identity, sexual orientation, or an intersection of these demographic factors.

Participants who identified as POC, LGBTQIA, and/or female characterized the current UWSPH climate as “somewhat uncomfortable” or “very uncomfortable” more often than their White, male, and non-LGBTQIA peers who often expressed a “very good” level of comfort. For underrepresented or minority students, their discomfort was linked to: (a) the perceived lack of academic, social, or psychological support for their groups; (b) the inability of faculty to genuinely connect with them in the absence of shared identities; (c) the lack of competency of faculty in addressing microaggressions, bias, racism, and sexual prejudice within the classroom setting; and (d) the lack of confidence in UWSPH leadership to resolve inequities that make the climate uncomfortable. This pattern was consistent with responses from faculty and staff in the same demographic groups. Their discomfort was linked to a lack of advocacy or allies resulting from very few POC, LGBTQIA, or women in faculty and leadership positions. Additionally, several respondents perceived structural oppressions based on race, ethnicity, and/or gender that required them to take on tasks that are not part of their position or prevented professional growth.

My role is not to be a secretary. But there have been many times where I have not been asked but told “You're taking notes today” or “You need to do this.” … sometimes, I wonder would they say it in a different way if other people were around or if I was not a person of color, a woman of color. (Staff, POC).

Some LGBTQIA- identifying respondents recounted the discomfort by their peers in having discussions around gender identities.

When the topic of like gender-neutral or non-binary pronouns come up… everyone wants to use the right pronoun, but people are so awkward about it. [I recall a colleague] who [also] uses gender-neutral pronouns, someone literally going through every pronoun that was not the right one until they got to the right one. For something that seems to be brought up so much… people are pretty not great at it. (Faculty, LGBTQIA).

The positive assessments of the UWSPH climate generally came from faculty, staff, or students who identified as White, especially from males, expressing that they have felt and continue to feel comfortable in UWSPH. Many of these participants acknowledged their positive assessment was likely linked to the benefits they receive from their racial or gendered privilege. Many White respondents also recognized that underrepresented groups often feel uncomfortable and could recall specific instances where POCs faced discrimination.

We had a front desk person here who was a Black man, and he had to go do an errand, I think, for… someone in a leadership position here. And [he] had to go talk to a faculty member who was a White woman of prestige. And I can't remember the details, but… they called security on him. I mean, he didn't do anything but go to deliver a piece of paper or get a piece of paper and was treated as a criminal. I mean, there are incidences like that [and] you recognize, ‘That wouldn't have happened to me.’ (Faculty, White).

2. The lack of diversity in UWSPH

One of the consistent sentiments that emerged across the demographic groups was that the UWSPH population lacks diversity. The need for a more diverse faculty body was especially important for students, who felt that the current teaching styles and perspectives did not provide enough emphasis on discussions around the importance of diversity or the lack of equity in public health research and practice. Among students, participants described that the racial diversity that exists in the UWSPH population was driven by the large population of international students, and that there are few POC from the United States. Some LGBTQIA faculty acknowledged the lack of representation of LGBTQIA professors and the constant burden of having to self-identify to their colleagues. However, they acknowledged the importance of being able to support LGBTQIA students by expressing their identities. Other faculty also acknowledged that the current mentorship system rewards the more affluent students and further marginalizes groups who come from cultures where they are not encouraged to approach their professors or feel uncomfortable doing so:

…[one thing] we've tried to be a little more intentional about in our research group is that usually we end up giving projects to people who come ask for them. And there's a very specific group of people who come and ask for projects. And a lot of people who would be great, or who I would enjoy mentoring don't come and ask. So, how do we find them and encourage them to come and get a project and have—and what kind of biases are we perpetuating by sort of continuing to work with the same group of people over and over? Or people who've had a certain type of education that makes them feel confident enough to ask for a project. (Faculty, Women).

3. UWSPH operates with an inherent structural hierarchy that perpetuates White privilege

The sentiment of limited diversity was grounded in observations that UW continues to be a “White university” with a “White culture,” and that programs or curricula are built for and around the experiences of the dominant racial group. As a result, there appeared to be an overwhelming perception that the backgrounds and experiences of POCs are not well-understood by faculty and school leaders. Faculty, staff, and students were overwhelmingly conscious that they operate within an inherent structural hierarchy in UWSPH. While such hierarchy was widely recognized as common to academic institutions—with some faculty and staff pronouncing that this structure will not change or that they are powerless to change it—there remained a general feeling that such hierarchy breeds discomfort and perpetuates either White privilege, male privilege, and/or class privilege.

So, in terms of leadership, there are a lot of White privileged men who have been here for quite some time and then, the bureaucracy in itself, it's an institution, right, that's been there forever… and the bureaucracy protects them… And so, for me as a staff at the level I am, I feel like perhaps I can grow but there's definitely a limit. And I think it depends on who I have connections with and my supervisor, again, what privileges they have and what kind of power they have in the organization and how much they're willing to bat for me because that is, eventually, going to be the catalyst that maybe supports some potential movement upwards. (Staff, POC).

Students often acknowledged that faculty still sent subconscious cues in terms of preference to acknowledge White students more than those of color when they get silenced in classes by not being called on or acknowledged by White faculty.

…every time I bring up sort of indigenous things [in class], or talking about indigenous rights, or how it incorporates into sort of population health… it gets seen as pseudoscience [by the professor]. She'll do this thing where White students will say stuff, she'll write it down because it's interesting, and she'll acknowledge that. But then whenever people of color talk in the class, it seems like she's not writing anything down. And that signals… in this conversation who you value, who you don't. (Student, POC).

Several staff participants also felt that these hierarchical structures caused them to stifle their identity at work and quiet their voice in the presence of colleagues with greater authority and power in order to avoid being perceived as “difficult” or “pushing too much for change.” For some POC staff, they generally seemed to feel that their race played a significant role in how they interact with White colleagues and felt more conscious of their racial identity and how they present themselves.

I think the way I show up to …work was the best representative of myself. I wanted to make sure that I set the tone for being one of the few… persons of color in the office. I felt like I needed to make sure my hair was combed, and I was dressed professionally and that I watched my language and made sure I didn't show too much of my personality… (Staff, POC).

Students also questioned the accountability for faculty considered as the aggressor during racially-oriented incidences, specifically detailing how power and privilege allowed for second chances despite the severity of situations.

I took a class …and the professor, who I think is close to retiring, he said something very racist in class and… no longer taught the class after that class occurred. But then… as they're creating this new curriculum, I think he's the head of two committees that's rewriting the curriculum for the class, right? And I just think that that says something about tenured professors. (Student, POC).

Additionally, for the distribution and access of key resources, the hierarchical system was perceived to favor those with a certain level of power and privilege, with POCs feeling fearful to ask for resources that they feel should also be made available to them, despite seeing them being made available exclusively to their White counterparts. In contrast, some White faculty respondents did not perceive any hierarchical system for promotions or access to resources. Instead, they felt that there were clear rules that were not impacted by identity.

I hadn't actually felt that being White was either an advantage or a disadvantage for me. I never felt like it made a difference with respect to promotion, for example. The rules were really clear. You publish this much, you get this much grant money—you know, you do these things. Didn't seem to me that it had anything whatsoever to do with any other of my characteristics. (Faculty, White)

### Positive Experiences Linked to Identity and Role Within UWSPH

When participants were asked to recount positive experiences related to their identity or role within UWSPH, they experienced satisfaction with and optimism for the trajectory of the UWSPH climate around the themes of: (a) a recognition of an increased commitment to improve the climate, (b) the observation of persons in power acknowledging their privilege, and (c) the active efforts around reducing issues related to racism, sexism, classism, heterosexism and transphobia through the current School- and University-wide DEI strategy.

1. An increased commitment to improve the climate

Generally, participants recognized increased commitment from faculty, staff, and students “to want to do better” and noted the increased prioritization of DEI across campus. There were reported instances of students receiving sufficient academic and personal support from faculty despite not having a shared identity in terms of race or gender. Additionally, there were increasing efforts to match students more appropriately with academic advisors who students could identify with beyond a shared research interest. Faculty respondents shared key steps they were taking to build diversity and inclusivity in their curriculum and pedagogy. For example, some professors began their courses with introductory sessions that include discussions of race, gender, and class and how those play out in the course material, while others started the course by acknowledging their privilege, or developing shared ground rules rooted in undoing racism, sexism, classism, and sexual prejudice. These efforts were not lost on students, as some respondents noted their support for professors who were intentionally taking active steps to build a safe space in the classroom.

From the 1st day of class, all the three instructors introduced with their pronouns, and even [recognized] when they were presenting with examples on papers that they found to be problematic. (Student, LGBTQIA).

2. Recognition of power and privilege among individuals in organizational positions of power

Some faculty members who occupy social and organizational positions of power were able to recognize their privilege, whether it be from their race, gender, sexual orientation, socio-economic class, or role.

This year, we added like a little opening talk about our own perspective as teachers. So, I said, “I'm a cisgender woman. I'm heterosexual. I grew up in a privileged background,” that kind of discussion; like recognizing my own privilege and then putting it out there as part of something that's okay to talk about as part of the perspectives we take in the class… I think that it opened up a tiny space for students to feel like, “Okay, your thoughts on these topics are welcome here.” It's not off-limits to go there. (Faculty, White).

3. Acknowledgment of structural efforts to enhance and promote diversity

A majority of participants were aware that a senior position focused on improving DEI was created in UWSPH, and that school- and department-wide DEI strategy documents exist. Despite feeling there was a lack of sufficient diversity within departments, faculty and staff felt that hiring efforts use a diversity framework, especially by involving departmental DEI committees within current hiring processes. Respondents also noted they observed more specific diversity-related events, including diversity workshops for faculty and staff, and student-center events led by student groups, which were perceived to create opportunities to further create a culture of inclusion.

### Negative Experiences Linked to Identity and Role Within UWSPH

Students, faculty and staff were also asked to recount negative experiences related to their identity or role within UWSPH. Key emerging themes were similar to those reflecting UWSPH's climate assessment, including: (a) race, ethnicity, gender identity, and sexual orientation as key drivers of negative experiences, (b) non-inclusive course content and curricula, (c) lack of competency among faculty to respond to issues concerning DEI, and (d) persistence of male privilege and misogynistic perceptions around gender roles and motherhood.

1. Race, ethnicity, gender, and sexual orientation as key drivers of negative experiences

An overwhelming majority of the negative experiences of faculty, staff, and students revolved around race, ethnicity, gender and/or sexual orientation. This perception was consistently observed among POC, who felt excluded, discriminated against, or made invisible in a “White-centered” environment. For students, the effect of these negative experiences ranged from emotional discomfort from constantly experiencing imposter syndrome, to, adverse effects on their learning and grades that made them walk out of class angry or consider dropping out. In some extreme cases, students recounted thoughts about harming themselves due to the extreme pressures and stress and leaving the program.

Also, just generally just dealing with having to be a person of color in this type of institution is just exhausting. It's just kind of tiring to have to constantly deal with these microaggressions and second-guessing yourself. It just takes up a lot of brain space, I think, and so sometimes after I've been in this type of situation, I'm just tired. I don't want to deal with anyone else anymore. (Student, POC).

For me, my 1st year, I actually got depressed and I…wasn't able to get [psychological help]…. I was… pretty much sleeping in the lab, trying to meet my—at the time—PI's expectations, and just being treated so bad. I had to leave school for 2 months and go back [home] to try to get out of depression… I actually was thinking of committing suicide… my work would be more productive if the lab environment could be more friendly and more inclusive. But it's the only way… to keep my career going until I graduate. (Student, POC).

For students and faculty/staff who identified as LGBTQIA, a common issue was the neglect of gender identity and sexual orientation in curricula or in interactions with peers.

…I think the thing that's come up on more than one occasion that's sort of odd is when people say, “Oh, we're still talking about pronouns?” Or have this mentality like, “Well, we talked about this at the last meeting, so why are we still?” And again, it's like a subtle thing, but to me, like you said this is about cultural change, and that takes time. It's not like you learn this in one meeting's worth of time… I think there's interest in it, but when it takes more than 30 min of brain space, it's like, “Well, that's too much.” (Faculty, LGBTQIA).

2. The curricula and course content across UWSPH are perceived as being exclusive of content featuring minoritized racial and ethnic groups, international populations, and LGBTQIA identities

Participants perceived that UWSPH programs, curricula, course materials and content were not reflective of the diverse academic experiences of students and their delivery is not inclusive of marginalized populations, including non-US citizens, racial and ethnic minorities, and non-heterosexual and non-cisgender identities. Specifically, students felt there is a very myopic approach to teaching public health in UWSPH, with some participants stating that the presentation of academic content is culturally insensitive or exclusive. They presented examples of how course texts, supplementary reading materials, or examples given in class discussions appeared to be relevant only to the American experience in public health, and primarily reflective of cisgendered, heteronormative, or outdated scholarship.

And there was another dataset where there were some individuals who had sex assigned at birth that was different than gender identity, and the professor chalked that up to being a data error. And it's like, or we could talk about gender minorities and how some people don't identify with the sex that they were assigned at birth… [There's] an opportunity to say, “Up to now, this isn't the standard. We normally don't ask sex and gender and differentiate, but moving forward, we should be talking about that.” And we should be talking about how race itself isn't really a determinant for most diseases; it's racism that is. (Student, LGBTQIA).

3. Faculty lack the competency to respond to issues concerning equity, diversity, and inclusion in the classroom

A common sentiment from faculty, staff, and students is that faculty are not adequately skilled to address exclusionary, divisive, or socio-politically charged situations in the moment when they occur in classrooms or other learning environments. Students overwhelmingly felt that faculty lack the cultural and theoretical competency to respond to moments when microagressions, inappropriate comments, or exclusionary behavior occur, or to generally present course material in an inclusive manner. Consequently, students who might be offended are left to either stay silent in fear of retaliation or to defend themselves or their group in the absence of faculty support.

…it shouldn't be the responsibility of the class, the students, to argue with another classmate if something really inappropriate does come up. And that frequently happens in my courses, where someone will say something that's just off the wall, and then I'm like, “Is the professor going to say anything?” And then they don't, and then I have to. And it's like, “I'm not getting paid to do this, so why am I left to be responsible for handling this inappropriate comment just as a person of color?” (Student, POC).

White faculty felt that differences in treatment based on race was a new observation for them and acknowledged inaction, or not knowing how to act, when their peers face discrimination. Some White faculty feel somewhat uncomfortable with the current UWSPH climate, stemming from the view that students are now more outspoken, vigilant, and less tolerant of micro- and macro-aggressions, which consequently makes them more anxious in their interactions with students for fear of being misinterpreted or poorly evaluated.

…students have changed. I'm not sure exactly why or how, but [they]… are much more sensitive to the power imbalances, to the race and ethnicity imbalances, to the dissonance between the school and the university stated values and what they see, physically, in the classroom. And I think they're much less tolerant of those variances and our excuses and our trying to explain away why things aren't different. (Faculty, White).

4. The existence of male privilege and misogynistic perceptions around gender roles and motherhood

Male privilege emerged as a prominent theme when discussing negative experiences of female-identifying respondents. Female faculty felt that they are more often than not passed up in terms of their professional growth and they are often expected to take on duties such as planning and organizing meetings.

…there are certain male professors who are my equivalent in rank and skill set, but who appear to be moving quickly through different things, for very unexplained reasons. And then, the second thing is, I think, the office housework concept… like why am I organizing all these meetings and arranging all these things and doing all this stuff? And the people that I consider my equals who are men don't seem to be doing that. They don't seem to have as many of those of those non-academic responsibilities. Or they seem to be able to say no to them more, without having consequences, whereas I would feel like I—I feel like I really have to do all those things. (Faculty, Women).

Women faculty, staff, and students also highlighted the occurrence of misogynistic behavior or microagressions from their male counterparts. This can be in the form of regularly being talked over at meetings or in class discussions, to receiving comments about their appearance. One major area in which male privilege and double standards based on gender became more palpable was parenthood. Participants felt that faculty and staff who are mothers were treated differently compared to those who are fathers or single. Additionally, there was also a common perception that motherhood limits career possibilities and scholarly productivity, especially around inequity of the provision of support for maternal roles.

When I announced that I was pregnant with my second child, my mentor—in front of other people—said, “Was it a failure of birth control? Because certainly, you would not have planned this.” It was amazingly inappropriate. (Faculty, Women).

Linked to the stigmatization of motherhood in academia was a profound disconnect or lack of empathy when it came to policies on maternity leave, childcare, child rearing, and how those matters might relate to the career trajectory of female faculty or staff.

### Recommendations and Top Priorities for Diversity, Equity, and Inclusion Efforts

Recommendations for how to improve the learning climate to combat inequitable, biased and exclusionary perspectives and behavior centered around four themes: (a) developing a robust program of continuous learning to promote DEI-related themes, such as recognizing the role of power and privilege in generating social inequities and undermining health, (b) redesigning and developing a more representative and inclusive curriculum, (c) intentionally diversifying faculty, staff, and the student population, and (d) providing more academic, professional, emotional, and psychological support for marginalized or underrepresented groups.

1. Develop DEI competency through a robust program of continuous learning

There was an overwhelming perception that faculty, staff, and students believe in the importance of UWSPH training on key fundamental DEI concepts, including racism; diversity; biases; micro- and macro-aggressions as related to race, class, and gender and sexual orientation. The necessity of workshops that promote training and learning on DEI for faculty and staff emerged as the top priority across all cohorts, given their perceived lack of ability to recognize and address situations in the moment when they arise.

… creating [a] cultural shift is not just about best practices. It's not just about understanding a concept and a training… To me when I think about what it means to be my best self is that I need to be able to reflect on my own thinking, my own biases, my own… I don't know. It's deeper work than just learning about what my core aggression is and how to hopefully not do it… I think the work it's going to take to shift the culture is really one of how do you get people to pause and look at their own selves enough, and deep enough, to not just get defensive? (Faculty, LGBTQIA).

A significant number of faculty, staff, and students recommended making these workshops mandatory, especially since these types of training are usually attended by POC staff. Making these trainings a requirement helps ensure that standard practices and behavior can be achieved especially in terms of classroom management and course delivery. However, some voices from the faculty and leadership were skeptical of mandatory training because of time constraints and demanding too much of faculty. Participants also suggested that new students undergo DEI-related training or orientation sessions, especially international students who may not be familiar with American culture, to prepare them for instances they might face based on their identities.

2. Redesign a more representative and inclusive curriculum

Developing an inclusive curriculum to curtail exclusionary behavior or discussions strongly emerged as another priority for faculty, staff, and students. Respondents defined an inclusive curriculum as one that: (a) highlights the experiences of marginalized and underrepresented groups and is responsive to the needs of all students by acknowledging the social justice aspects of public health curriculum; (b) moves away from a US-centric foundation to a more global one; (c) has equitable performance measures for students; and (d) provides opportunities for more inclusive and meaningful learning regardless of a student's background or identity. Some students felt that not enough attention was paid to student evaluation to improve courses.

I'm a rep on …the curriculum committee for [REDACTED] and they look at all the courses and just quickly look at mean scores. And if it falls below a satisfactory, a three, then they'll flag it to be like, “We should come back and talk about this briefly.” But that's it—it's very, very minimal… They don't look at the qualitative [feedback] at all. (Student, White/Women).

3. Diversify faculty, staff, and the student populations

There was a general view that intentionally diversifying the UWSPH population—students, faculty, staff, and leadership—would improve the overall climate by making it more inclusive, safe, and comfortable. Specifically, there were suggestions to hire and retain more faculty and staff of color, women and LGBTQIA-identified individuals.

Well, most of my professors are just old White men. I think that diversifying the professors would go a really long way, even if they don't change anything in the curriculum because I think that professors of color will bring a different perspective and talk about equity and things in a different way that would be helpful. (Student, POC).

4. Provide academic, professional, emotional, and psychological support for marginalized or underrepresented groups

Participants identifying as part of a marginalized group, specifically POCs, LGBTQIA, and women—and those whose identities intersect multiple marginalized groups–felt the need for more academic, professional, and emotional or psychological support.

Throughout my graduate education, I've kind of been told that like, “Make sure you exercise. Make sure you take space if you need it” …And I just realized that, yes, they are helpful, but what I really need to do is to talk to someone because… I can do all these things, but still at the end of the day when I get home, I'm just so exhausted and tired. And I just realized why that was, and it was because [the things recommended were] not necessarily what I need. (Student, POC).

This need was also acknowledged by participants from dominant groups who benefit from certain types of privilege by virtue of being White, male, or heterosexual. Suggestions for provision of support included: (a) creating more opportunities for underrepresented and/or marginalized students to collaborate and/or network with faculty, advisors, or other students and build community; (b) improving mentorship opportunities for underrepresented students by not only matching them to faculty based on their academic and research interests, but also identifying additional mentors with shared identities, including race/ethnicity, sexual orientation, language or place of origin to form a mentorship team; (c) creating more awareness of psychological support catered to underrepresented groups; (d) strengthening policies on parental leave and childcare and providing emotional and professional resources for parents (including student-parents); and (e) strengthening the use and normalization of gender-inclusive language.

## Discussion

In this study, we aimed to capture and summarize the perspectives of UWSPH faculty, staff and students regarding their experiences and assessment of the learning climate at the school. Although UWSPH faculty, staff, and students could clearly define their ideal climate and had a collective recognition of an increased commitment to improve the climate, the experiences of respondents revealed the ongoing dissonance between the school's DEI mission and lived experiences. Findings suggest that people of minoritized identities, in terms of their race, ethnicity, gender, and sexual orientation, are often uncomfortable and experience overt and covert negative experiences related to their identities. For these groups, the lack of diversity at UWSPH perpetuates a structural hierarchy favoring White, male, and heteronormative privilege.

Our findings align with previous research indicating that institutions that permanently uphold White ideology have hostile environments for minority and underrepresented students, consequently leading them to have poorer learning outcomes than their counterparts (11–14). Similar to findings from a recent multi-institutional qualitative study (15), POCs in our study felt excluded, discriminated against, or invisible in a White- or Western-centered environment and curriculum. Their White counterparts recognized how their privilege may have blinded them to the differences in treatment from their peers, despite being aware of isolated incidents. This sentiment of “White blindness obscures and protects White identities,” especially within institutional curricula by making race a prohibited or awkward subject, defining racism as something of the past, and developing skeptic responses to experiences of minority groups (11, 16). This restriction yields an erasure or downplay of minority issues and supports the racial and social hierarchies that perpetuate discrimination (11).

Women often highlighted misogynistic behavior from their male counterparts, by describing how academic/professional opportunities can be restricted and how motherhood is stigmatized and seen as a barrier. They commonly expressed concern over their vulnerability when their male counterparts continue to operate from a place of power and when the structure of UWSPH continues to support such social hierarchy. This notion aligns with the scholarly work examining the roles of women in higher education that conveys that within institutions, women predominantly play “housekeeping roles,” which simultaneously places them within the “managerial yet marginalized fabric of the organization (17).” Other research suggests that women who work in academia often have negative equity due to the burden placed on them by society to balance their roles in the home and place of employment, forcing them to practice bias avoidance, where they often have to hide their familial obligations for career growth (18–20). Female faculty respondents recalled instances of feeling uncomfortable when announcing their pregnancies or needing to leave meetings early to manage child care and other family obligations.

For LGBTQIA-identifying participants, issues relating to gender identity and sexual orientation continue not to be given adequate attention, specifically regarding discussing and addressing exclusionary or insensitive research or course materials. Numerous other studies conducted on college campuses mirror these experiences, detailing both direct and indirect aggressions and emphasizing the importance of recognizing and understanding the differences amongst LGBTQIA student experiences in order in developing inclusive and effective strategies to improve the learning climate (21–24). Respondents who identified as LGBTQIA recalled a variety of negative experiences that reflected the different dimensions of their sexual and gender identities, including: discrimination based their physical presentation, frustration with being called the incorrect pronoun, fear of disclosing their identity, and discomfort discussing their intimate relationships or bringing their partners to campus events.

The wide range of negative experiences from our participants reflects those in institutions across the country, which points to rampant neglect to understand the effects of racism, White privilege, patriarchy, heterosexism and transphobia within HEIs. The burden of naming these issues and developing solutions should be placed on those with power and privilege, especially institutional leadership who drive the vision for their schools and departments. Yet, the marginalized continue to bear the brunt of the weight, furthering their burden. This study highlights the importance of distinguishing the experiences and perspectives of various populations within academic institutions in order to equitably understand and meet their needs and it attempts to give those in power actionable items to work toward equity. Acting on the priorities suggested by our participants will directly combat the occurrence of biases, micro- and macroaggressions and address the lack of inclusivity in our teaching curricula by focusing on developing and improving competency across UWSPH through training; curriculum redesign; population diversification; and provision of academic, professional, emotional, and psychological support, especially for marginalized or underrepresented groups. These improvements are necessary as HEIs should be held accountable for establishing a climate that promotes and protects its entire population by welcoming diversity, supporting equity, and promoting inclusion. The suggestions brought forth are supported by and included in the Transformational Tapestry Model (25), a transformation strategy for HEIs based on organizational change theory that defines climate as being essential in the improvement process. For transformation to be sustainable, it is important that institutions who conduct these climate assessments tie recommendations to specific objectives, acknowledge the continuous, non-linear, nature of institutional change as it refers to climate transformation, and ensures the intentional involvement of key stakeholders involved in the transformation process (26). One key to success will be the development of an operational plan with specific evaluation metrics for climate improvement and other metrics that directly or tangentially assess students' learning outcomes. This plan needs to be accompanied by sufficient funds to support the actions and periodic measurement of the metrics. Woodard and Sim's programmatic plan may be useful for the sustainable operationalization of these suggestions, as it transitions from smaller interventions on the cognitive and social level such as signatures of commitment, learning sessions, and cultural activities into larger cultural and systematic shifts, including bias and attitudes work, development of support system, and curriculum changes (26).

Additionally, it is critical that institutions do not implement strategies for the sake of merely increasing tolerance. Institutions must be invested in sustainable, systematic change by challenging the dominant ideology and dismantling the systems that enact and normalize them. HEIs must combat the aforementioned factors that maintain negative climates, especially for students. Otherwise, they will continue to perpetuate the harmful experiences detailed by our respondents, further impacting their mental and psychological well-being. Long term consequences of poorly guided actions also can lead to adverse academic outcomes and further widening of the achievement gap (24, 26, 27).

This work indicates the importance and value of robust qualitative research to assess the learning climate as it reveals the lived experiences of communities within higher education institutions, especially among those within underrepresented and marginalized populations who often have negative experience related to their identities. A major strength of this study is the disaggregated data that provide insights into experiences from a variety of population groups that mirror the heterogeneity of most higher education institutions. One limitation stems from our participants, as volunteer respondents are likely to be those who are already engaged or interested in DEI-related issues and therefore, we may have missed the perspectives and experiences of those who are less interested or knowledgeable in DEI, which may be a key audience for developing a conducive learning climate. Additionally, at recruitment, we did not collect individual-level data on the unique identities of our participants, which did not allow for us to enumerate the various types of sub-identities and intersecting identities within our interview groups. A major challenge during recruitment was the difficulty of capturing a sufficient number of certain identities. Future studies should ensure they maximize representation across all identities to allow for deeper and intersectional analyses.

Findings from our study illustrate the importance of assessing the interaction of structural, psychological, and behavioral aspects of the teaching, mentorship and learning experience within HEIs through the lived experiences of their communities. Additionally, we elicited recommendations for enhancing the learning climate and driving sustainable institutional change. Individuals who hold privilege based on their role, identity, or positionality must first recognize their privilege and make themselves aware of the various types of harm occurring in their institutions. This recognition means being able to reflect on the ways that their social status—race, gender, or class–has given these privileged identities a certain advantage because of structural oppressions and how the system perpetuates these oppressions; therefore, they must use these advantages to take direct action to prevent the perpetuation of any form of exclusion. In order to develop and maintain an environment that drives student success, academic institutions, including UWSPH, must not merely be interested in addressing issues related to DEI, but intentionally invested and committed to developing a climate that places anti-racism, anti-sexism, anti-classism, anti-heterosexism and anti-transphobia as an institutional priority and provides sufficient support and resources to drive impactful and sustainable progress.

## Data Availability Statement

The raw data supporting the conclusions of this article will be made available by the authors, without undue reservation.

## Ethics Statement

The University of Washington Human Studies Division determined that this study was minimal risk and exempted from ethical review. The identities of all participants were only known to the FGD facilitators during the recruitment process and participants remained anonymous during data collection. No reference to their names, titles, or roles were made during the FGDs. If such references were accidentally broached during the discussion, these names or titles were redacted during the transcription process. All FGD audio files were stored in a password-protected cloud storage system accessed only by the immediate research team. In order to ensure confidentiality for all participants, all names and identifying characteristics have been anonymized.

## Author Contributions

All authors listed have made a substantial, direct and intellectual contribution to the work, and approved it for publication.

## Conflict of Interest

The authors declare that the research was conducted in the absence of any commercial or financial relationships that could be construed as a potential conflict of interest.
