# Supplementary material for: “Being a Person of Color in This Institution Is Exhausting”: Defining and Optimizing the Learning Climate to Support Diversity, Equity, and Inclusion at the University of Washington School of Public Health
Source: Front Public Health. 2021 Apr 15;9:642477. doi: 10.3389/fpubh.2021.642477 (PMC8082071; doi:10.3389/fpubh.2021.642477)
Supplement: Supplementary file 1 [file Table_1.DOCX]

Supplementary Material

# Supplementary Table 1: Codebook

| PRIMARY CODE | SECONDARY CODE | TERTIARY CODE | DESCRIPTION |
| --- | --- | --- | --- |
| Being Faculty |  |  | Participant teaches courses at SPH |
| Being LGBTQ+ |  |  | Participant identifies as LGBTQIA+ |
|  | I feel I’m coming out all the time |  | As LGBTQIA, participant feels the need to explain or defend their gender identity to others at SPH |
| Being POC |  |  | Participant identifies as a person of color or mixed race individual |
| Being Staff |  |  | Participant works as non-teaching or administrative personnel in SPH |
| Being White |  |  | Participant identifies as white (non-person of color) |
|  | I don’t fit into many of the bins |  | As a white person, feeling excluded from diversity affinity groups |
|  | I felt I was the problem for someone |  | As a white person, feeling that he/she was bringing discomfort to another albeit unconsciously |
|  | Being oblivious |  | As a white person, being oblivious to the plight or experiences of underrepresented groups in SPH |
|  | White privilege |  | The automatic privileges or benefits that accrue to a person by simply being white |
|  | White guilt from faculty |  | As a white faculty member, feeling individual or collective guilt for harm resulting from racist treatment of underrepresented groups in SPH by other white people both historically and currently |
| Being a School Leader |  |  | Participant holds leadership position in SPH |
|  | Efforts at building ideal climate |  | As a school leader, feels that there are various efforts being undertaken to build an ideal climate in SPH |
|  |  | Communication and openness | Belief of school leader that communication and openness need to be top priorities of school leaders with regard to DEI issues |
|  |  | Creating “structural things” | Efforts by school leaders to improve climate by designing and implementing structural (i.e., systemic, or policy) changes in SPH |
|  |  | Equal pay for equal work | Addressing pay equity/equal pay-for equal work issues for women faculty and staff |
|  |  | Leading by doing | As a school leader, feels that improving climate in the context of DEI depends largely on having school leaders directly engaging in DEI activities, attending events, and strategy implementation |
| Being a Woman |  |  | Participant identifies as a woman |
|  | I’m conscious of the way I interact |  | As a woman faculty, staff, or student, the person is conscious of how she speaks, interacts, or behaves because of stereotypes based on her gender |
|  | Double standards |  | Women and men holding the same positions or roles are treated and/or evaluated following different standards based on gender |
|  | Female faculty evaluated more harshly |  | Female faculty observe that they are more harshly evaluated formally and informally by students |
|  | Male privilege |  | The automatic privileges or benefits that accrue to a person by simply being male |
|  | Men talk over you |  | Women experiencing being regularly talked over or interrupted during meetings or class discussions |
|  | More men in leadership |  | There are more men holding positions of leadership or authority in SPH |
|  | Motherhood as a disadvantage |  | Female faculty, staff, and students, observe that motherhood is seen as a professional disadvantage in academia or in any professional workplace |
|  | We’re treated “second to men” |  | Women feel that they are treated second to men |
| Being a Student |  |  | Participant is an undergraduate or graduate student in SPH |
| Importance of ideal climate |  |  | Questions on how important an ideal learning and working climate (in general) is for participants |
|  | Essential |  | An ideal climate is essential to learning and working optimally |
|  |  | Climate sets tone for belongingness | An ideal climate sets the tone for belongingness |
|  |  | Optimal for curricular engagement | An ideal climate is optimal for students to engage with the curriculum and achieve positive learning outcomes |
|  | Not Important |  | An ideal climate is not important to learning and working optimally |
|  | Very Important |  | An ideal climate is very important (but not essential) to learning and working optimally |
| Imposter Syndrome |  |  | The feeling where there is doubt about one’s accomplishments and a persistent internalized fear of being exposed as a "fraud;" in academia, feeling that one doesn’t belong because of perceived inadequacies despite credentials or capabilities |
| Marital Status |  |  | Marital status as a source of discrimination of exclusion |
|  | Being married as career limitation |  | Perception that being married limits one’s potential or career possibilities in SPH |
|  | Marital status social divide |  | In SPH, the sense of people working better with or socializing with people with the same marital status, creating cliques |
| Negative Experiences |  |  | Negative experiences in SPH |
|  | Students are very sensitive |  | Faculty feel that students are increasingly very sensitive about race, gender, class issues; very politically correct |
|  | Condescension |  | Experiencing condescending behavior or treatment from others |
|  | Curriculum not inclusive |  | SPH curriculum is perceived as not inclusive or exclusionary in terms content and delivery referring to race, class, and gender |
|  |  | Content doesn’t reflect our values | Course content perceived as not reflective of what SPH stands for |
|  |  | Content presentation not DEI sensitive | Course material is not presented in a manner sensitive to DEI |
|  |  | Fat-shaming | Experiencing health content and presentation of material that shames fat or obese people |
|  | Faculty-student disconnect |  | The experienced disconnect between faculty and students in SPH |
|  |  | Gender disconnect | Faculty don’t seem to understand gender identity issues |
|  |  | Large class size | Class sizes are too large to provide meaningful discussions or mentor relationships |
|  |  | Difficulty establishing relationships | The distance between faculty and students is wide and there’s no opportunity to establish academic/mentor relationships |
|  |  | Unclear expectations | Teachers do not set clear expectations for student performance or output |
|  | Feeling unsafe |  | Participant feels unsafe in SPH physically, emotionally, or psychologically |
|  |  | Anxious while teaching | Faculty feel anxious while teaching in the classroom for fear of being misinterpreted |
|  |  | Devaluation of individual epistemologies | Experiences and beliefs of a person are devalued or sidelined |
|  |  | Fear of judgment | Feeling unsafe because of fear that they will be judged for what they say, do, or believe |
|  |  | Fear of punishment or retaliation | Feeling unsafe because of fear that they will be punished for what they say, do, or believe, or experience retaliation when they speak up against something or someone |
|  |  | It’s a “macro thing” | Reference to the larger socio-political climate external to the university or SPH |
|  |  | Negative effects on mental health | Feeling unsafe because of negative effects on one’s mental health |
|  |  | Physically unsafe | Feeling physically unsafe in SPH, fear of bodily harm |
|  |  | Scared to speak up | Feeling scared to speak up against something or someone that causes discomfort or offence |
|  | Gender identity issues |  | Negative experiences related to gender identity issues |
|  |  | Lack of LGBTQ representation | Perception that there are very few persons who identify as LGBTQIA in positions of leadership or who are faculty |
|  |  | Use of pronouns | Using gender sensitive pronouns as an issue of inclusion |
|  | Inability to respond to increased social awareness of students |  | Perception that faculty members are not able to or do not have the cultural competence to respond to/address socially or politically sensitive issues that come up in class, or respond to increased social awareness of students |
|  |  | Faculty lack the competency | Students or teachers feel they do not have the cultural competency to deal with DEI issues and need more training on that |
|  |  | Generational gap | The generational gap between faculty and students creates an uncomfortable climate |
|  |  | No time for training | Faculty do not have extra time to attend DEI training sessions |
|  | Inappropriate behavior/comments |  | Inappropriate behavior/comments regarding race, gender, class, etc. within a learning environment |
|  |  | Exclusionary behavior | Behavior of an individual or group that excludes others, particularly underrepresented groups |
|  |  | Political differences | Differences or disagreements due to political or ideological differences |
|  | Instance of discrimination |  | Instances when people experience discrimination based on the race, gender, sexual orientation, or socio-economic status |
|  |  | Discriminatory behavior | Instances when people felt discriminated based on their race, gender, sexual orientation, or social status |
|  | Issue is under the radar |  | Perception that DEI issues are not being discussed or addressed, or that particular issues for one group are not recognized by school leaders or the community |
|  |  | No sense of community around issues | Perception that the SPH community is not addressing a certain DEI issue |
|  |  | Not enough discussion | Perception that the SPH community is not talking about concerns or issues about DEI |
|  | Lack of academic support |  | Perception that students are not receiving adequate academic support in SPH |
|  |  | Funding | Need for more funding options and opportunities for students |
|  |  | Need for mentorship | Students feel the need for more mentorship from faculty they directly work with |
|  |  | Need more professional opportunities | Students feel the need for more professional opportunities in research or on the field related to their areas of specialization |
|  | Lack of accessibility |  | Perception that SPH is not accessible in terms of physical accessibility |
|  |  | People are literally far apart | Facilities and offices are separated, which does not make it conducive for community building |
|  | Lack of diversity among faculty/staff |  | Perception that faculty and staff in SPH are not diverse and representative of minorities |
|  | Lack of parity |  | Perception that there is no equality of treatment among groups, especially regarding status or pay. |
|  | Lack of respect for staff |  | Staff feel that they are not respected by their managers or persons of authority |
|  | Lack of understanding |  | Perception that the experiences of minority groups are misunderstood or unappreciated |
|  | Microagressions |  | Brief and commonplace daily verbal, behavioral, or environmental indignities, whether intentional or unintentional, that communicate hostile, derogatory, or negative prejudicial slights and insults toward any group, particularly culturally marginalized groups. |
|  |  | “It’s just exhausting” | Marginalized groups feeling emotionally exhausted by having to deal with microagressions or exclusionary behavior on a regular basis |
|  | Misogyny |  | Dislike of, contempt for, or ingrained prejudice against women |
|  | No credit where credit is due |  | Feeling that one is not being given credit by managers or faculty for work done |
|  | Not Diverse |  | Perception that SPH does not have a diverse population |
|  |  | “It’s a different culture: it’s white” | Perception that the SPH culture and climate is geared toward or designed by and for white people |
|  |  | “Only a climate of liberalism” | Perception that SPH only subscribes to a liberal or progressive political ideology to the exclusion of others, particularly conservatism/Republicanism |
|  |  | Can’t identify with faculty/advisor | Students feeling that they cannot identify with their faculty/advisor because of race or gender |
|  |  | Faculty/staff not diverse | Perception that faculty and staff are not representative of marginalized groups, and are mostly white |
|  |  | Not enough representation in leadership | Perception that school leaders are still mostly white men and need to be more diverse |
|  |  | Tokenism in hiring/admissions | Perception that when persons of color, women, or LGBTQIA are hired, that they are hired because of the need for a “quota” but not because of a sincere intention to diversify |
|  | Race/Ethnicity issues |  | Negative experiences rooted in race or ethnicity |
|  |  | “They didn’t know they were black until they came here” | International students from Africa who did not feel excluded because of racism against blacks until they came to the United States |
|  |  | International students seem invisible | International students feel that they are excluded by faculty and peers in SPH |
|  |  | Cultural divide creates tensions | Differences in culture and racial or ethnic identities create divisions and tensions |
|  |  | Language barrier creates disconnect | International students feel excluded in their learning environment because of the language barrier |
|  |  | POC faculty disrespected by students | Instances when faculty of color were observed to be disrespected by students |
|  |  | POCs are invisible | Persons of color feeling excluded in SPH |
|  |  | Tokenism | The practice of making only symbolic effort to do a particular thing, especially by recruiting a small number of people from underrepresented groups in order to give the appearance of sexual or racial equality within a workforce. |
|  |  | White centered programs | Perception that SPH academic programs are designed by and for white persons, effectively excluding the needs and experiences of underrepresented groups |
|  | Sexual misconduct |  | Negative experiences due to sexual misconduct of others |
|  | Structural hierarchy |  | Organizational or structural hierarchy in SPH that is perceived to perpetuate social status based on race and gender |
|  |  | “No one actually listens” | Feeling that school leaders and faculty do not genuinely pay attention to DEI concerns |
|  |  | Faculty/leadership defensive | Perception that faculty and school leadership become defensive when faced with DEI issues against faculty, staff, or leadership |
|  |  | Power differential due to gender | Persons of authority having power based on gender, where males have more power of others |
|  |  | Power issues between faculty | Faculty having power issues based on seniority, race, or gender |
|  | Work-Life balance not valued |  | Faculty or staff feeling too stressed in the workplace |
|  |  | No flexibility | Staff work schedules are not flexible, which promotes poor work-life balance |
| Positive experiences |  |  | Positive experiences in SPH |
|  | Commitment to wanting to do better |  | Perception that school leaders, faculty, staff, and/or students want to do better in terms of addressing DEI issues |
|  |  | Guidance from faculty | Some faculty are currently providing good mentorship or academic support for students |
|  |  | Improving course delivery | Some faculty are trying to improve course delivery in terms of inclusiveness |
|  |  | Staff are welcoming to students | Students feel that staff are welcoming to them |
|  |  | Support from faculty | Students feel that there is adequate academic support from faculty |
|  | Recruitment considering on DEI |  | Perception that diversity, equity and inclusion are considerations in hiring |
|  | Classroom experience |  | Perception that the classroom environment is comfortable and pleasant |
|  |  | Dialogue opportunities with professor | Presence of opportunities to dialogue with the professor during class and outside class |
|  | Diversity |  | Positive experiences with regard to diversity in the learning environment |
|  |  | “They’re younger” | Perception that young faculty and staff are being recruited |
|  |  | Availability of support resources | Minority groups feel that there are resources provided to support their learning or working success |
|  |  | Diversity strategy exists | Awareness that an EDI strategy exists in the school |
|  |  | Effort to hire more minorities in leadership | SPH is doing more to hire minorities in leadership positions |
|  |  | Faculty are in tune with minorities | Perception that teachers understand the needs of minority students |
|  |  | Hiring POC faculty and staff | Perception that more persons of color are being hired in faculty and staff positions |
|  |  | Starting to discuss EDI issues | Perception that more members of the SPH community are discussing and addressing EDI issues |
|  |  | We have more Diversity Events | Perception that there are more events and activities to promote inclusion, and celebrate diversity |
|  | Equity |  | Positive experiences related to equity |
|  |  | Equal pay for equal work | School leaders say that there is progress being made on pay equity based on gender |
|  |  | Financial aid for underrepresented groups | There are increasing available financial aid options for minority students |
|  |  | Recognizing privileges | Persons who do not identify with minority groups acknowledge their privilege and make efforts to address inequity issues |
|  | Inclusion |  | Positive experiences related to inclusion/inclusiveness |
|  |  | Inclusive course delivery | Perception that there are efforts to make course delivery inclusive |
|  |  | Lifting others' voices | Efforts to give minority groups a voice; making sure that minority groups are represented in discussions or decision-making |
|  |  | Opportunities to get to know each other | There are opportunities to make more connections or relationships at SPH |
|  |  | People are warm | Perception that people at SPH are warm and welcoming |
|  |  | Student commitment | Perception that there are students committed to addressing EDI issues |
|  | Safety |  | Positive experiences related to safety or feeling safe |
|  |  | Support and connection with peers | Feeling that there is support from and connection with peers |
| Recommended Priorities |  |  | Recommended priority areas in reference to EDI issues from faculty, staff, students, and school leaders |
|  | Academic support |  | There should be academic support for all students, especially underrepresented groups |
|  |  | Mentorship | There is a need for strong mentorship between faculty and students |
|  | Mental health support |  | There is a need for mental health support, especially for students |
|  | Clarity of expectations and roles |  | There should be clarity of expectations from teachers, and clarity of roles of staff vis-à-vis managers/administrators |
|  | Cultural change |  | There is a need for cultural change around EDI issues |
|  |  | “Humility and accountability” | School leaders, faculty, or those with authority must be held accountable and practice humility when faced with EDI issues or complaints |
|  |  | Acknowledge power of faculty vs. staff | The influence or power of faculty over staff has to be acknowledged |
|  |  | DEI education for students | There is a need for DEI education/training for students |
|  |  | Intentional efforts to build community | Efforts to build inclusiveness and community have to be intentional |
|  |  | More support for international students | There needs to be more academic and emotional support for international students |
|  |  | More support for LGBTQ community | There needs to be more academic and emotional support for the LGBTQ+ community |
|  |  | More support for students who are parents | There needs to be more academic and emotional support for students who are parents |
|  |  | More support for women faculty, staff, and students | There needs to be more academic and emotional support for women faculty, staff, and students |
|  |  | Programs must be all-inclusive | Academic programs must be inclusive of all groups |
|  |  | Talk about issues | There needs to be discussion of EDI issues |
|  | Curricular development |  | Recommendations related to curricular development |
|  |  | Acknowledging socio-political aspects of PH curriculum | The public health curriculum needs to be reflective of the socio-political aspects of the field in theory and practice |
|  |  | Global' curriculum (not-US centric) | The public health curriculum needs to be more global in presentation rather than focused on the Western or US experience |
|  |  | Have equitable performance measures | The curriculum needs to have equitable performance measures |
|  |  | Incorporating more POC material | The curriculum needs to incorporate more texts, research and course material authored by persons of color or other minority groups |
|  |  | Integration of DEI concepts | The curriculum needs to incorporate EDI concepts and be inclusive |
|  |  | More opportunities for active learning | The curriculum needs to provide more opportunities for active learning |
|  |  | Responding to academic needs of all students | The curriculum needs to respond to the academic needs of all students, especially minorities |
|  | Diversity in recruitment |  | Recommendations related to increasing diversity in recruitment |
|  |  | Diversity in hiring | There needs to be more diversity in recruitment and hiring of faculty and staff |
|  |  | Diversity in admissions | There needs to be more diversity in recruitment and admissions of students |
|  |  | Having people look like them makes a difference | Perception that having people who look like students or who they can identify with makes a difference in their learning success/outcomes |
|  |  | POC faculty retention | There is a need to focus on efforts to retain persons of faculty once they are hired |
|  | Leadership |  | Recommendations related to school leadership |
|  |  | Active leadership in DEI | There is a need for school leaders to be more active in addressing EDI issues and implementing the EDI strategy across SPH |
|  |  | Diversity in leadership | There is a need to diversify SPH leadership and make it more representative |
|  |  | Financial support for DEI efforts | There needs to be more funding for EDI programs |
|  |  | More support for faculty training | There needs to be more funding and resources for faculty EDI training |
|  |  | Providing internal support resources | There needs to be more internal support resources for minority groups |
|  |  | Formulate solutions | School leaders need to formulate tangible solutions beyond the overarching EDI strategy |
|  |  | Improve top-down communication | Improve feedback process, increase awareness of available resources |
|  |  | More transparency with equity issues | School leaders need to be more transparent with SPH faculty, staff, and students about how they address equity issues |
|  |  | Streamline DEI efforts | There is a need to streamline all EDI efforts across SPH departments |
|  | More DEI training for faculty/staff |  | More regular EDI training is needed for faculty and staff |
|  |  | Develop cultural competency | Cultural competency needs to be developed, especially among faculty and staff |
|  |  | Evidence-based best practices | Evidence-based best practices in EDI should be adopted by SPH to feed into the EDI strategy |
|  |  | Knowing how to present course material | Faculty must have the competency to present course material in an inclusive manner |
|  |  | Make DEI training mandatory | Perception that EDI training needs to be mandatory |
|  |  | MIcroagression training | EDI Training needs to focus on microagression training |
|  |  | More conversations about issues | There is a need for more conversations about EDI issues among different groups in SPH |
|  |  | Gender identity training | There is a need for gender identity competency, and knowledge of how to navigate gender identities professional spaces |
|  |  | More holistic approach | There is a need for a more holistic approach to EDI training |
|  |  | More modes for training | EDI training must be available to faculty, staff, and students through different modes |
|  |  | Racial competency | There is a need for more racial competency among faculty, staff, and students in SPH |
|  |  | Recognizing privilege | People who hold privilege based on race, class, gender need to recognize and check their privilege |
|  |  | Sustained effort | EDI training has to be regular and sustained, not just a one-time event |
|  | Regularly measure climate |  | The SPH climate needs to be regularly measured |
|  | Reviewing diversity in admissions |  | Diversity must be reviewed and considered in the student admission process |
|  |  | Active recruitment of diverse prospective students | There must be active recruitment of prospective students from minority groups |
|  |  | Conversations about race and ethnicity in admissions | School leaders and admissions committee(s) must have a thorough understanding of and conversations about race and ethnicity as considerations for admissions |
| Socio-economic status |  |  | Socio-economic status as an EDI issues |
|  | Financial insecurity leads to discomfort |  | Financial insecurity among students leads to discomfort within the learning climate |
| “Ideal” Climate |  |  | How participants defined their ideal learning or working climate in general |
|  | Nurtures growth |  | A climate that nurtures academic and professional growth |
|  |  | Students motivated to learn | A climate where students are motivated to learn and take risks |
|  | Comfort |  | Feeling of comfort |
|  |  | Freedom to ask questions | Having the freedom to ask questions inside and outside the classroom |
|  |  | Freedom to make mistakes/take risks | Having the freedom to make mistakes and take risks in one’s learning or working environment |
|  | Conducive to free expression |  | A climate that is conducive to free expression inside and outside the classroom |
|  |  | Can communicate openly | A climate that allows people to communicate openly without judgment |
|  |  | Can challenge assumptions | A climate where people can challenge assumptions about norms and issues |
|  |  | People’s thoughts are valued | A climate that values people’s thoughts regardless of background |
|  | Diversity |  | A climate that values diversity of people and cultures |
|  |  | Professors aware of privilege | Faculty are aware of their privilege based on race, gender, socio-economic class, or position/authority |
|  |  | Representation matters | A climate where representation among faculty, staff, students and school leaders matters, especially in positions of authority |
|  |  | Seeing people like you | A climate that makes it possible for one to see people who look like you or who you can identify with based on race, gender, or class |
|  | Effort to make things happen |  | A climate where people make an effort to make things happen in terms of equity, diversity, and inclusion |
|  |  | Data-driven decision making | A climate that is shaped by decision-making processes that are data-driven |
|  |  | Transparency from leadership | A climate where school leaders are transparent in their decision-making and how they handle EDI issues |
|  | Ensuring meaningful learning |  | A climate where faculty and staff ensure meaningful learning for students |
|  | Faculty |  | Characteristics of an ideal climate related to faculty |
|  |  | Approachable faculty | A learning climate where students find faculty to be approachable |
|  |  | Expert instructors | A learning climate where faculty are experts in their field and are capable instructors |
|  |  | Strong mentorship | A learning climate that has a strong mentorship program or opportunities for students to be mentored effectively by faculty or advisers |
|  |  | Teachers trained in DEI | A learning climate that is supported by teachers trained to handle or address diversity, equity, and inclusion issues in the classroom |
|  | Inclusion |  | A climate that values inclusion |
|  |  | Encourages collaboration | A learning and working climate that encourages research or professional collaboration across departments |
|  |  | Inclusive curriculum | A learning climate that is shaped by an inclusive and responsive curriculum |
|  |  | Shared participation and interaction | A climate where students share experiences, participate, interact inside and outside the classroom |
|  |  | Trust | A climate where there is trust between and among all members of the community |
|  | Physical space |  | Characteristics of an ideal climate related to physical space |
|  |  | Small class sizes | A learning climate that values small class sizes |
|  |  | Conducive facilities | The existence of facilities conducive for learning or that provide venues for effective and robust learning experiences |
|  | Respect |  | A climate where there is mutual respect |
|  | Safety |  | A climate where people feel safe physically and/or emotionally |
|  |  | No verbal attacks | A climate that feels safe because there are no verbal attacks or microagressions against others that threaten safety |
|  |  | Physical safety | A climate wherein people feel safe physically |
|  |  | Psychological support | A climate that feels safe because it provides avenues and resources for psychological support |
|  | Thoughtful reflection |  | A climate that encourages thoughtful reflection among its members especially with regard to diversity, equity, and inclusion |
| Rating of SPH Climate |  |  | How participants assessed the learning or working climate of SPH (based on a loose rating scale of 1 to 5 to serve as a starting point for discussion, with 1 being very uncomfortable and 5 being very comfortable). This was a qualitative assessment, and participants could use descriptors beyond the scale to qualify their response) |
|  | "It's a White university" |  | Perception that UW is a university with a predominantly white population, a climate that is shaped by white experiences, and a curriculum that is focused mostly on white students |
|  |  | Faculty not diverse/representative | Perception that faculty are mostly white and not representative of students of color, women, or other minority groups |
|  | "Students have changed" |  | Perceptions (from faculty) that students have changed in that they are more socially and politically aware or active; less tolerant of DEI infractions |
|  | Climate not good |  | Perception that the SPH climate is “not good” |
|  |  | “There are people who haven’t talked to me” | Feeling ostracized and alone in one’s learning and working climate |
|  |  | Behind on supporting women in maternal roles | Perception that SPH is behind in creating and implementing policies that support women in maternal roles, e.g. maternity leave, breastfeeding |
|  |  | Faculty fear being misinterpreted | Professors feel anxious in their interactions with students in and out of the classroom for fear of being misinterpreted when they present course material or address issues of race, gender, or class during discussions |
|  |  | Need for faculty training | Learning climate is not good because faculty need to have DEI training to address DEI issues in class |
|  |  | Not comfortable | Perception that the learning or working climate is “not comfortable” |
|  |  | Somewhat uncomfortable | Perception that the learning or working climate is “somewhat comfortable” |
|  |  | Very uncomfortable | Perception that the learning or working climate is “very comfortable” |
|  |  | People are not speaking up | Perception that students, staff, and some faculty are not speaking up when faced with DEI issues |
|  | Emphasis on self-care |  | SPH learning and working climate seems to emphasize self-care rather than community support |
|  | Good but needs improvement |  | Perception that the learning or working climate is good but needs improvement |
|  |  | “Good for me not for others” | Persons who do not identify with minority groups realize that the climate may be good for them, but not for others |
|  |  | “I’m used to compromising” | Persons of color stating that they are used to compromising in terms of expecting a comfortable or ideal learning climate |
|  |  | Inclusiveness is ongoing struggle | Perception that the climate is good for the most part but inclusiveness is an ongoing struggle |
|  |  | Somewhat comfortable | Perception that the learning or working environment is “somewhat comfortable” |
|  |  | There has been growth | Perception that there has been progress in learning or working climate in terms of DEI but that there is more to be done |
|  |  | Disconnect between words and actions | Perception that there is a disconnect between what school leaders say the DEI policy/strategy is and the actions taken to address concerns related to diversity, equity, and inclusion |
|  | Very comfortable |  | Perception that the learning or working climate is “very comfortable” |
|  | Lack in confidence in leadership |  | Lack in confidence that school leaders will adequately address DEI issues, especially those concerning discrimination |
|  |  | Posturing by leadership | Perception that there is only a lot of posturing by school leaders, with no real action |
|  |  | No culture of feedback | Perception that there is no culture of feedback and addressing feedback in SPH |
|  |  | No support | Perception that there is no support from school leaders on DEI issues |
|  |  | Structure and hierarchy based on privilege | Difficult to make changes because of a structure and hierarchy based on privilege, particularly white privilege and male privilege |
|  |  | Unclear about where to take problems | Students and staff feel unclear about where to take their problems related to diversity, equity, and inclusion |
|  |  | Unsure of whether issues will be resolved | Unsure of whether school leaders will resolve complaints related to DEI issues |
|  | Neutral |  | Feeling neutral about the current SPH climate |
|  | Resources |  | Responses related to SPH resources that shape the learning and working climate |
|  |  | Doing a good job of providing resources | Perception that SPH is doing a good job in providing resources to support DEI programs/the EDI Strategy |
|  |  | Lack of awareness of resources | Students seem to lack awareness of support resources related to DEI issues |
|  | Staff not “seen” |  | Staff feel that they are not given proper regard and credit for what they do; they feel invisible and forgotten especially when it comes to professional development or promotions |
|  | Change in expectations |  | Perception that expectations have changed in a faculty-student relationship and that this change hasn’t been made clear |
