# Supplementary material for: “Being a Person of Color in This Institution Is Exhausting”: Defining and Optimizing the Learning Climate to Support Diversity, Equity, and Inclusion at the University of Washington School of Public Health
Source: Front Public Health. 2021 Apr 15;9:642477. doi: 10.3389/fpubh.2021.642477 (PMC8082071; doi:10.3389/fpubh.2021.642477)
Supplement: Supplementary file 2 [file Table_2.DOCX]

Supplementary Material

# Supplementary Table 2:

All quotes, perceptions of UWSPH populations on learning climate by study domains and themes. Additional quotes not included in the body of the manuscript are in bold

| **THEME** | **SUPPORTING QUOTES** | |
| --- | --- | --- |
| **Domain 1:** **The ideal climate and its importance to learning and working** | | |
| The ideal learning climate | “[Our] experience should be challenging and rigorous from an academic standpoint, but … [interactions with our teachers or peers] shouldn't necessarily add to that difficulty. It should be facilitating learning and not adding to some type of traumatic experience that the student then has to process through.” (Student, POC) | |
|  | “Something else that is really important to the learning climate is also just making sure that the people who are here do feel like they belong. And I think that a big part of that is really understanding how the decision makers are implementing policies and creating policies and their transparency around different choices that they’re making.” (Student, Women) | |
|  | “I think my ideal learning climate…is an environment where everyone feels comfortable and empowered to have discussions, ask hard questions, and feel like we're all progressing together.” (Student, Women) | |
|  | “[The climate] has to be respectful bidirectionally, comfortable – meaning you can make mistakes and take risks.” (Faculty, Leadership) | |
|  | “Another thing that I think greatly impacts the learning climate, is the physical space where classrooms take place… I know that it’s difficult for the university to figure out that schedule and everything but really, having enough physical space to be comfortable in a room, and have discussions, and not be packed in like sardines, I think is important.” (Student, Women) | |
| The impact of climate comfort on learning outcomes | - “I think if you could have professors who make you uncomfortable or don't make you feel safe, you [can] succeed in the class by just getting the grade that you need to get. I don't think that it puts you in a position to really succeed outside of the classroom. You're not gonna reach out to the professor for mentorship; you're just gonna go to the class and go home.” (Student, POC) | |
|  | - Out of 120 students this year …we failed one student and felt terrible about that because it was a student of color…and we reached out repeatedly. I will never know what failed for that student. I know we failed because she didn't complete the class. I have to believe a part of it was the environment.” (Faculty, White) | |
| **Domain 2:** **Assessment of the current UWSPH climate** | | |
| Race and gender identities and sexual orientation have a big impact on one’s comfort within the climate at UWSPH. | | “My role is not to be a secretary. But there have been many times where I have not been asked but told ‘You’re taking notes today’ or ‘You need to do this.’ … sometimes, I wonder would they say it in a different way if other people were around or if I was not a person of color, a woman of color.” (Staff, POC) |
|  |  | “When the topic of like gender-neutral or nonbinary pronouns come up…everyone wants to use the right pronoun, but people are so awkward about it. [I recall a colleague] who [also] uses gender-neutral pronouns, someone literally going through every pronoun that was not the right one until they got to the right one. For something that seems to be brought up so much… people are pretty not great at it." (Faculty, LGBTQIA) |
|  |  | “We had a front desk person here who was a Black man, and he had to go do an errand, I think, for … someone in a leadership position here. And [he] had to go talk to a faculty member who was a White woman of prestige. And I can't remember the details, but…they called security on him. I mean, he didn't do anything but go to deliver a piece of paper or get a piece of paper and was treated as a criminal. I mean, there are incidences like that [and] you recognize, ‘That wouldn't have happened to me.’" (Faculty, White) |
|  |  | “[Women are] second to men. I think one of the basic best examples of microaggression [in the] learning climate in this institution is the way men speak over women and speak first, and it’s so apparent [everywhere]…There’s no pause to give voice to anyone else…and that [also] comes out in our students.” (Faculty, Women) |
|  |  | “I appreciate that there are others within the department that acknowledge… that I’m a person of color that is taken advantage of but what are you going to do [about it]?” (Staff, POC) |
|  |  | “I was at the SPH website today going through the diversity portion of it but then, I clicked leadership and then, that’s where I saw the lack of diversity at least in terms of race and ethnicity. So, I think thinking about that, that’s why I feel just neutral, in general…I think, at the end of the day, it just seems like if there is not representation from higher levels that the comfort is just okay.” (Staff, POC) |
| UWSPH lacks diversity | | “…[one thing] we’ve tried to be a little more intentional about in our research group is that usually we end up giving projects to people who come ask for them. And there’s a very specific group of people who come and ask for projects. And a lot of people who would be great, or who I would enjoy mentoring don’t come and ask. So, how do we find them and encourage them to come and get a project and have – and what kind of biases are we perpetuating by sort of continuing to work with the same group of people over and over? Or people who’ve had a certain type of education that makes them feel confident enough to ask for a project.” (Faculty, Women) |
|  |  | “… I recently had a friend that was doing something with some youth that were interested in global health. And there was a [POC] student there that was from south Seattle, and she was saying that ‘Nothing like this is ever advertised to us’… we do have a lot of international students, but most of the students that are from the U.S. are White. And I think that just outreach, like reaching out to those communities within the U.S. is equally as important.” (Student, POC). |
|  |  | “I feel like I do a lot of work – I feel like I'm constantly coming out, which is also exhausting to be doing it all the time. But I also feel like it’s super important, like I have an outward facing picture of me and my wife. It's very clear that I'm a queer faculty member and that's been particularly important for students that I've met with who are queer as a place where we can connect…not only in physical representation, but also in discussions.” (Faculty, LGBTQIA) |
|  |  | But I honestly feel like I got [hired] for those skills but also because the Department of [redacted] was very white and they need diversity. And so, they’re allowing staff roles to be diverse but, again, those faculty roles, not so much. And I think that’s because to keep the power and the way things have been done here the same. But also…to meet a quota… of saying, okay, we do have people of color on our staff.” (Staff, POC). |
| UWSPH operates with an inherent structural hierarchy that perpetuates White privilege | | “So, in terms of leadership, there are a lot of White privileged men who have been here for quite some time and then, the bureaucracy in itself, it’s an institution, right, that’s been there forever…and the bureaucracy protects them…And so, for me as a staff at the level I am, I feel like perhaps I can grow but there’s definitely a limit. And I think it depends on who I have connections with and my supervisor, again, what privileges they have and what kind of power they have in the organization and how much they’re willing to bat for me because that is, eventually, going to be the catalyst that maybe supports some potential movement upwards.” (Staff, POC) |
|  |  | “…every time I bring up sort of indigenous things [in class], or talking about indigenous rights, or how it incorporates into sort of population health…it gets seen as pseudoscience [by the professor]. She’ll do this thing where White students will say stuff, she’ll write it down because it’s interesting, and she’ll acknowledge that. But then whenever people of color talk in the class, it seems like she’s not writing anything down. And that signals… in this conversation who you value, who you don’t.” (Student, POC). |
|  |  | I think the way I show up to …work was the best representative of myself. I wanted to make sure that I set the tone for being one of the few … persons of color in the office. I felt like I needed to make sure my hair was combed, and I was dressed professionally and that I watched my language and made sure I didn’t show too much of my personality…” (Staff, POC) |
|  |  | “I took a class…and the professor, who I think is close to retiring, he said something very racist in class and … no longer taught the class after that class occurred. But then…as they’re creating this new curriculum, I think he's the head of two committees that's rewriting the curriculum for the class, right? And I just think that that says something about tenured professors. (Student, POC). |
|  |  | “I hadn't actually felt that being white was either an advantage or a disadvantage for me. I never felt like it made a difference with respect to promotion, for example. The rules were really clear. You publish this much, you get this much grant money – you know, you do these things. Didn't seem to me that it had anything whatsoever to do with any other of my characteristics.” (Faculty, White) |
|  |  | “I sometimes feel like as a woman of color [that] I get silenced a lot sometimes in classes. Like either it'll be a discussion … and I'll say one thing, and then I'm not allowed to say anything else, which I think is…kind of discriminatory because maybe I have expertise on the subject but I'm not allowed to speak anymore. And it's not that anyone sits there and says, ‘You can't talk anymore," but I just won't get called on…” (Student, POC). |
|  |  | **“The reason why I don’t speak up is because I don’t want to be perceived as… the one that’s pushing too much for change or the one that has all of these different ideas or that’s difficult because I think the way to move up in this type of environment, in my opinion, is to conform. So, that means losing a piece of my identity, of my voice, of the things that I strongly feel passionate about, especially when it comes to working with people of color. I’ve only been here for a short amount of time and I already know my role. I know that when this person is in the room, I need to stay in my space, in my spot. Don’t talk too much. Don’t have an opinion.” (Staff, POC)** |
|  |  | **“I start to second guess myself ‘should I ask for what I want and need or do I have to wait or do I have to bring out the policy that says I can do it?’. I don’t feel comfortable just asking for some of the things that I want and need but I have seen that other people that are White have…those things [made] easily accessible [for them].” (Staff, POC)** |
|  |  | **“I have had an experience in class where a guest lecturer, who is still a faculty member in the School of Public Health came in to talk about cocaine-exposed infants. And …for half of the presentation, she was calling them "crack babies" and putting up pictures of Black and Brown babies. And myself and another student consistently raised our hands, asking where her research was from, why she was using those articles, why she was using those photos, why she was using that language. And she kept saying that she would get to it later in the presentation, and then she took a break …and the student next to me went up to her and said very loudly so a lot of people could hear, ‘Why aren't you addressing our questions?’ She didn't say it in an aggressive way, but we had been asking questions, and the professor just kept saying, ‘Later, later, later.’ So, she just wanted to know why, and the professor got really defensive. And then when she continued with the presentation, she changed her language to "cocaine-exposed infants" [but never answered our questions].” (Student, POC).** |
| **Domain 3:** **Positive experiences linked to identity & role within UWSPH** | | |
| Commitment to improve the climate is growing | “From the first day of class, all the three instructors introduced with their pronouns, and even [recognized] when they were presenting with examples on papers that they found to be problematic.” (Student, LGBTQIA) | |
|  | “I think there definitely is an uptick of conversation and action around creating a more inclusive learning environment around diversity. UW at large put out a diversity roadmap. I know the school and departments have been working on how do we incorporate that in, not only to faculty recruitment, staff recruitment, and just everyday culture.” (Faculty, Women) | |
|  | “She [a professor] acknowledges when students bring in diverse perspectives that don’t agree with evidence…Everything is so evidence-based in public health. Everything is so science-based, right? Well, it’s like people have lived experiences too that they bring into the classroom, right? And let’s acknowledge that and maybe put it in conversation…about why that is.” (Student, White) | |
| There is growing recognition of power and privilege among individuals in leadership positions | “This year, we added like a little opening talk about our own perspective as teachers. So, I said, ‘I’m a cisgender woman. I’m heterosexual. I grew up in a privileged background,’ that kind of discussion; like recognizing my own privilege and then putting it out there as part of something that’s okay to talk about as part of the perspectives we take in the class… I think that it opened up a tiny space for students to feel like, ‘Okay, your thoughts on these topics are welcome here.’ It’s not off-limits to go there.” (Faculty, White) | |
|  | **“Some of the faculty at [REDACTED] Department, even though they are old White men, I like that they kind of recognize that and acknowledge it. And they're like, ‘It is who I am. I can't really change where I'm coming from, but this is my perspective with all this privilege that I do have.’ And so, I do appreciate that they kind of acknowledge it, at least.” (Student, White)** | |
|  | **“So, this is not in the classroom, but in my research work, which is still a learning environment. I am fortunate where I am one of numerous people of color – women of color, actually – and our PI and my advisor is a white male. One of my co-students/colleagues was going to be giving a talk about racism and being a person of color in academia, and [our PI] sent an email specifically to all of us talking about how important it was and how much we should be there to support, and I thought that was very good on him – and that he would be there, and I thought that was very good on him as a white male to be there.” (Student, POC)** | |
| There are strategic and active efforts focused on enhancing and promoting diversity | **“So, when I first started, there weren’t a lot of people of color. I think I was one of the first of the few staff members in this space that we’re in right now that was hired. And then, I’ve noticed progressively there have been more people of color that have been showing up as staff, not as faculty. We have had a few faculties that have been hired that are people of color, which is really nice to see.” (Staff, POC)** | |
| **Domain 4:** **Negative Experiences Linked to Identity & Role within UWSPH** | | |
| Race, ethnicity, gender, and sexual orientation are key drivers of negative experiences | “Also, just generally just dealing with having to be a person of color in this type of institution is just exhausting. It's just kind of tiring to have to constantly deal with these microaggressions and second-guessing yourself. It just takes up a lot of brain space, I think, and so sometimes after I've been in this type of situation, I'm just tired. I don't want to deal with anyone else anymore.” (Student, POC) | |
|  | “For me, my first year, I actually got depressed and I…wasn’t able to get [psychological help]….I was…pretty much sleeping in the lab, trying to meet my – at the time – PI’s expectations, and just being treated so bad. I had to leave school for two months and go back [home] to try to get out of depression…I actually was thinking of committing suicide…my work would be more productive if the lab environment could be more friendly and more inclusive. But it’s the only way…to keep my career going until I graduate.” (Student, POC) | |
|  | “…I think the thing that's come up on more than one occasion that's sort of odd is when people say, ‘Oh, we're still talking about pronouns?’ Or have this mentality like, ‘Well, we talked about this at the last meeting, so why are we still?’ And again, it's like a subtle thing, but to me, like you said this is about cultural change, and that takes time. It's not like you learn this in one meeting's worth of time…I think there's interest in it, but when it takes more than 30 minutes of brain space, it's like, ‘Well, that's too much.’" (Faculty, LGBTQIA) | |
|  | **I was the only Hispanic person in my class and I was so afraid, and everyone was judgmental, that it was just so hard to feel comfortable and safe... [or] to even pay attention to what was being said...and feel enthusiastic about learning. (Student, POC)** | |
|  | **“And there was another dataset where there were some individuals who had sex assigned at birth that was different than gender identity, and the professor chalked that up to being a data error. And it's like, or we could talk about gender minorities and how some people don't identify with the sex that they were assigned at birth… [There’s] an opportunity to say, ‘Up to now, this isn't the standard. We normally don't ask sex and gender and differentiate, but moving forward, we should be talking about that.’ And we should be talking about how race itself isn't really a determinant for most diseases; it's racism that is." (Student, LGBTQIA)** | |
| The curricula and course content across UWSPH are perceived to be lacking content that features minoritized racial and ethnic groups, international populations, and LGBTQIA identities | “…it shouldn't be the responsibility of the class, the students, to argue with another classmate if something really inappropriate does come up. And that frequently happens in my courses, where someone will say something that's just off the wall, and then I'm like, ‘Is the professor going to say anything?’ And then they don't, and then I have to. And it's like, ‘I'm not getting paid to do this, so why am I left to be responsible for handling this inappropriate comment just as a person of color?’" (Student, POC) | |
|  | **“In terms of Pacific Islander health… there are all these readings that have the category aggregated with API (Asian Pacific Islanders) … but I’m like, our health disparities are so different from East Asian people, kind of similar to Southeast Asian people, but they’re still different and they follow different narratives. And there’s indigenous people who are Pacific Islander, or Oceanic, that don’t get acknowledged in that space often either.” (Student, POC)** | |
|  | **“…something that frustrated me in my biostats classes is continually, we’re given this example about how you know – when you’re checking your data, how you know the data’s incorrect. The classic example is always given “someone who’s male, but pregnant.” That’s the classic example of what must be an incorrect data point. And, while that may be true for – most of the population is cisgender, I feel like there’s so many examples you could give that may marginalize the few students in the class who do identify [as transgender].” (Student, LGBTQIA)** | |
|  | **“… I was really disappointed with how the TA and the professor [of one of my classes] were kind of approaching teaching the subject matter and also creating the projects. Because it really favored people who had English as a first language. And just speaking with some of my classmates, they really struggled with the assignments that they had and it really hurt their GPA. The TA was kind of very nitpicky about grading on grammar and things like that instead of actual content…And so, there wasn’t really time to find support, especially if English wasn’t your first language.” (Student, White)** | |
| Faculty lack the competency to respond to issues concerning equity, diversity, and inclusion in the classroom | “…students have changed. I’m not sure exactly why or how, but [they]… are much more sensitive to the power imbalances, to the race and ethnicity imbalances, to the dissonance between the school and the university stated values and what they see, physically, in the classroom. And I think they’re much less tolerant of those variances and our excuses and our trying to explain away why things aren’t different. (Faculty, White) | |
|  | - **So, I’ve actually been called, ‘unfocused’ by several of my mentors for wanting to connect the dots to make it relevant to me and bring back to my communities. Because I think the focus is just about numbers. This is the foundation of public health. I understand that, but to remove how it’s connected to why there is such a lack of diversity that I have experienced, seems to be sweeping the problem under the rug.** | |
|  | “I think that the levels of concern have never been higher. I think the students are concerned. And I think the faculty are concerned. And I think faculty – I think particularly White male faculty feel on edge.” (Faculty, White) | |
|  | “…there are so many faculty [who]…really don’t know how to handle situations that are coming up. And not only do they not handle them well, but they are apprehensive — and, talk about fear — fearful of when it’s my turn to have to deal with something like this, and what will I do. (Faculty, White) | |
|  | “I've become more aware as, I think, many of us have over the past decade or more that our privilege has made us somewhat blind to what other [people] of color, women, have experienced. And, you know, I was just unaware of that.” (Faculty, White) | |
|  | So, I teach a pretty big undergraduate class and we talk about – one of the things we talk about – is the HIV epidemic here in the United States. And one of the things I taught them in class was that the first cases of HIV were among these group of four H’s – populations – heroin users, homosexuals, hemophiliacs, and Haitian immigrants. And it’s the same way ever year. And one of my students came up to me after class one day and said, “I was really interested in this, and I took it to one of my sociology classes so that I could kind of have parallel discussion, and I told my class about it, and my professor said that you’re a racist.” Because of the Haitian thing. And I was like – honestly, what I thought was – thank God he said this to me just one-on-one and that he didn’t say this to me in front of everybody. (Faculty, White) | |
|  | “…there are certain male professors who are my equivalent in rank and skill set, but who appear to be moving quickly through different things, for very unexplained reasons. And then, the second thing is, I think, the office housework concept…like why am I organizing all these meetings and arranging all these things and doing all this stuff? And the people that I consider my equals who are men don’t seem to be doing that. They don’t seem to have as many of those of those non-academic responsibilities. Or they seem to be able to say no to them more, without having consequences, whereas I would feel like I – I feel like I really have to do all those things.” (Faculty, Women) | |
| Male privilege and misogynistic perceptions around gender roles and motherhood persist | “When I announced that I was pregnant with my second child, my mentor – in front of other people – said, ‘Was it a failure of birth control? Because certainly, you would not have planned this.’ It was amazingly inappropriate.” (Faculty, Women) | |
|  | **“… We are so behind in this school and this department. A colleague of mine had a child, and there’s no private breast-pumping room in this building, and the solution was to walk across the street … to a random room and pump three or four times a day. It’s the equivalent of 45 minutes each time, just insane, just no awareness of that.” (Staff, Women)** | |
| **Domain 5:** **Recommendations and top priorities for diversity, equity, and inclusion efforts** | | |
| Develop DEI competency through a robust program of continuous learning | “… creating [a] cultural shift is not just about best practices. It's not just about understanding a concept and a training…To me when I think about what it means to be my best self is that I need to be able to reflect on my own thinking, my own biases, my own… I don't know. It's deeper work than just learning about what my core aggression is and how to hopefully not do it…I think the work it’s going to take to shift the culture is really one of how do you get people to pause and look at their own selves enough, and deep enough, to not just get defensive?” (Faculty, LGBTQIA) | |
|  | **“…we used to have diversity trainings but it was mostly an observation. It was mostly staff people. There are people of color who attended these. And it was like should we make it mandatory for all faculty, especially those higher ups and the leadership teams? Because you never really saw leadership or faculty members attending these diversity trainings.” (Staff, POC)** | |
| Redesign the curriculum to be representative and inclusive | I’m a rep on …the curriculum committee for [REDACTED] and they look at all the courses and just quickly look at mean scores. And if it falls below a satisfactory, a three, then they’ll flag it to be like, “We should come back and talk about this briefly.” But that’s it – it’s very, very minimal… They don’t look at the qualitative at all.” (Student, Women) | |
|  | **“And curriculum [also impacts the safety of the climate]. The syllabus, basically, is the first thing that people engage with, in the classroom. And when the syllabus is a certain way, or coming from only a certain point of view, or certain positionality, it kind of throws off the entire learning climate for the entire semester.” (Student, POC)** | |
|  | **“Some professors or some representatives are more able to kind of pretend that they’re listening to your opinion…when I tried to bring up some concerns [about the curriculum] and I just received like a, “Why are you so against change?” kind of response. And I felt kind of attacked when I tried to make these comments that I have brought up before. [The faculty are] just very politically answered…It seems like there’s this façade that they want feedback, but they have made these decisions already. And so, why bother?” (Student, White/Women)** | |
| Diversify faculty, staff, and the student populations | Well, most of my professors are just old White men. I think that diversifying the professors would go a really long way, even if they don't change anything in the curriculum because I think that professors of color will bring a different perspective and talk about equity and things in a different way that would be helpful. (Student, POC) | |
| Provide academic, professional, emotional, and psychological support for marginalized or underrepresented groups | Throughout my graduate education, I've kind of been told that like, ‘Make sure you exercise. Make sure you take space if you need it’ …And I just realized that, yes, they are helpful, but what I really need to do is to talk to someone because…I can do all these things, but still at the end of the day when I get home, I'm just so exhausted and tired. And I just realized why that was, and it was because [the things recommended were] not necessarily what I need.” (Student, POC) | |
